# Atorvastatin-loaded micelles with bone-targeted ligand for the treatment of osteoporosis

**DOI:** 10.1080/10717544.2017.1347966

**Published:** 2017-07-13

**Authors:** Yonghui Xie, Xueying Tan, Jian Huang, Hongwei Huang, Ping Zou, Jingbo Hu

**Affiliations:** a Department of Orthopaedics, Yangjiang People’s Hospital, Yangjiang, China;; b College of Pharmacy, Zhejiang Pharmaceutical College, Ningbo, China;; c Department of Pharmacy, The Central Hospital of Wuhan, Tongji Medical College, Huazhong University of Science and Technology, Wuhan, China;; d College of Pharmaceutical Sciences, Zhejiang University, Hangzhou, China

**Keywords:** Atorvastatin, bone-targeted, tetracycline, osteoporosis, micelles

## Abstract

Osteoporosis is a common bone disorder where the declined bone mass is far more than normal physiological status and usually associated with enhanced fracture risk, reduced bone strength and even deteriorated quality of life. Recent studies showed that statins could exert beneficial effects on bones via promoting osteoblastic activity mediated by increased expression of bone morphogenetic protein 2 and also by suppressing osteoclast proliferation. In this study, we developed atorvastatin-loaded tetracycline-poly (ethylene glycol)-poly(lactic-co-glycolic acid) (TC-PEG-PLGA/ATO) micelles for the targeted treatment of osteoporosis. The TC-PEG-PLGA was synthesized under the action of coupling reagents and then ATO was encapsulated through solvent diffusion method with encapsulation efficiency and drug loading of 89.32 ± 2.48% and 8.20 ± 0.53%, respectively. The release of ATO from micelles could be maintained for more than 48 h in pH 7.4 PBS. Pharmacokinetic results further demonstrated that TC-PEG-PLGA micelles could effectively shield ATO leakage from micelles and prolong their circulation time. Benefiting from TC specifically binding to hydroxyapatite (HAp), TC-PEG-PLGA/ATO micelles exerted good bone-targeted ability, as demonstrated by *in vitro* HAp affinity assay and biodistribution. Pharmacodynamic studies showed that TC-PEG-PLGA/ATO micelles could effectively improve bone mineral density and bone mechanical strength in osteoporotic rats. These results suggest that TC-PEG-PLGA/ATO micelles hold significant promise for the targeted treatment of osteoporosis.

## Introduction

Osteoporosis is a common bone disorder where the declined bone mass is far more than normal physiological status and causes severe disability to the patients, especially for the aged population (Lips et al., 2009). It is established that approximately two hundred million people suffer with osteoporosis around the world (Hamrick et al., 2015). Osteoporosis is usually associated with enhanced fracture risk, reduced bone strength and even deteriorated quality of life (Lips et al., 2009; Luhmann et al., 2012). Effective treatment of osteoporosis contributes to improve health care and reduces public health burden.

Recently available agents applied to osteoporosis treatment act on either the osteoclast via suppressing bone resorption or the osteoblast via stimulating bone formation (Shirke et al., 2008). Statins [3-hydroxy-3-methylglutaryl coenzyme A (HMG-CoA) reductase inhibitors] are extensively used for cholesterol-lowering treatment in clinical practice. In addition to lipid-lowering, they can also be used to stabilize atherosclerotic plaques, exert antioxidation, suppress inflammatory responses and regulate the immune function (Aukrust et al., 2005; Chan et al., 2013). In recent years, it has been demonstrated that statins exert beneficial effects on bone via promoting osteoblastic activity mediated by increased expression of bone morphogenetic protein 2 and suppressing osteoclast proliferation (Davignon & Mabile, 2001; Chuengsamarn et al., 2010; Esposito et al., 2013). However, long-term administration is often undesirable due to several side and out of target effects (Patel et al., 2016). In addition, they are limited by poor solubility and low bioavailability, which leads to the impracticable systematic administration in the clinical practice. Previous solutions included impregnation and repeated local injection, but the former could not determine the required dose, and the latter was easy to affect the healing process in the bone defect position and increased the risk of contamination (Shuid et al., 2013; Oryan et al., 2012; Moshiri et al., 2014; Oryan et al., 2014). Therefore, it is a pressing need to develop new treatments with enhanced therapeutic outcome and high safety.

Targeted drug delivery is a promising strategy to strengthen pharmacological effect and meanwhile minimize the side effects (Luhmann et al., 2012). The amphiphilic micelles-mediated by bone-targeted functional ligand may provide alternative solution at much lower doses and increase drug accumulated in bone tissues. The amphiphilic group spontaneously forms micelles with a core-shell structure in aqueous medium. Their hydrophobic group can store statins and the hydrophilic shell can guarantee their stability in an aqueous medium, the characteristics of which provides the large development potential used as drug carriers. The micellar delivery system modified by bone-targeted functional group represents a more effective and safe therapy method in comparison to impregnation and repeated local injections and hence cause strong interest in research.

Typical requirements for nano-drug delivery system for targeted therapy include: (1) prolonged circulation time; (2) increased accumulation of drugs in target sites; (3) protection of drugs from enzymatic and chemical degradation (Lips et al., 2009). Here, we aim to develop the amphiphilic copolymer poly (ethylene glycol)-poly(lactic-co-glycolic acid) (PEG-PLGA) micelles modified with tetracycline (TC) that is a well-known functional ligand to encapsulate hydrophobic atorvastatin (ATO) for the targeted treatment of osteoporosis. Both PEG and PLGA are biocompatible, which is extremely suitable for drug delivery system (Nicolas et al., 2013). Tetracycline (TC) is a broad-spectrum antibiotic used to treat a number of bacterial infections. Based on clinical findings, TC can specifically bond to hydroxyapatite Ca_10_(PO_4_)_6_(OH)_2_, the main components of bone tissue (Perrin, 1965). Accordingly, TC is good candidate for bone-targeted ligand. We assess the therapeutic effects of ATO-loaded TC-PEG-PLGA (TC-PEG-PLGA/ATO) micelles in an ovariectomy-induced osteoporosis rats. We also investigate micellar characteristics, cytotoxicity, cellular uptake mechanisms, affinity to hydroxyapatite, *in vivo* targeting ability and pharmacokinetic behaviors.

## Material and methods

### Materials

Atorvastatin was purchased from Energy Chemical Reagent Co, Ltd. (Shanghai, China); Tetracycline and Dicyclohexylcar-bodiimide (DCC) were obtained from Shanghai Aladdin Bio-chem Technology Co. Limited (Shanghai, China); PEG (HO-PEG-COOH, MW = 2000) was purchased from JenKem (Beijing, China); 4-Dimethylaminopyridine (4-DMAP) was purchased from Thermo Fisher Scientific (Waltham, MA); N-hydroxysuccinimide(NHS), poly(D,L-lactide-co-glycolide) (PLGA, L/G = 50/50, MW = 5000), pyrene, β-Glycerophosphate, L-ascorbic acid, and alizarin red S were purchased from Sigma-Aldrich (St Louis, MO); Hydroxyapatite (HAp) powder was purchased from Chemical Reagent Co, Ltd. (Shanghai, China); All other solvents and reagents were chemical grade.

### Synthesis of TC-PEG-PLGA

Firstly, PEG-PLGA was synthesized by esterification reaction between hydroxy group of PEG and carboxyl group of PLGA (1:1, mol:mol) in the presence of DCC and DMAP. Briefly, PLGA, DCC and DMAP (PLGA:DCC:DMAP = 1:3:0.3, mol:mol:mol) were dissolved in 30 mL anhydrous dimethyl sulfoxide (DMSO). The solution was stirred at 60 °C for 1 h to activate the carboxylic acid of PLGA under the protection of nitrogen. After PEG was added, the reaction was stirred at 60 °C under the protection of nitrogen for 24 h at 300 rpm. The reaction solution was then dialyzed (MWCO 7.0 kDa) t against pure water for 48 h with frequent exchanges of pure water to remove water-soluable byproducts. The dialyzed solution was centrifugated at 12 000 rpm to remove water-insoluble byproducts and lyophilized to achieve PEG-PLGA.

Next, TC-PEG-PLGA was synthesized via amide reaction between carboxyl group of PEG and amino group of TC in the presence of DCC and NHS. Similarly, PEG-PLGA, DCC and NHS (PEG-PLGA conjugates:DCC:NHS = 1:3:3, mol:mol:mol) were dissolved in 30 mL anhydrous DMSO. The solution was stirred at 60 °C for 1 h to activate the carboxylic acid of PEG-PLGA under the protection of nitrogen. After TC (TC: EG-PLGA = 1:1) was added, the reaction was stirred at 60 °C under the protection of nitrogen for 24 h at 300 rpm. The reaction solution was then dialyzed (MWCO 7.0 kDa) t against pure water for 48 h with frequent exchanges of pure water. The dialyzed solution was centrifugated at 12,000 rpm to remove water-insoluble byproducts and lyophilized to achieve TC-PEG-PLGA.

### Characterization of TC-PEG-PLGA

The composition of obtained TC-PEG-PLGA was confirmed by ^1^H NMR spectra on a Bruker (AVACE) AV-500 spectrometer. 20 mg Ml^−1^ TC, PEG, PLGA and TC-PEG-PLGA in dimethylsulfoxide-d_6_ were measured, respectively. The critical micelle concentration (CMC) value of TC-PEG-PLGA was determined by fluorescence measurement using pyrene as a probe (Du et al., 2010). The fluorescence spectra were recorded on a fluorescence spectrophotometer. The excitation wavelength was set at 337 nm and the pyrene emission was monitored at wavelength range of 360–450 nm. The concentration of TC-PEG-PLGA solution containing 5.94 × 10^−7^ M of pyrene varied from 1.0 × 10^−3^ to 1.0 mg mL^−1^. From the pyrene emission spectra, the intensity ratio of the first peak (I_1_, 374 nm) to the third peak (I_3_, 385 nm) was calculated for the determination of CMC value.

### Preparation of ATO-loaded TC-PEG-PLGA micelles

The ATO-loaded TC-PEG-PLGA (TC-PEG-PLGA/ATO) micelles were prepared through solvent diffusion method. ATO methanol solution (1 mg ml^−1^) was dropwise added to TC-PEG-PLGA solution (1 mg ml^−1^) under the treatment of probe-type ultrasonication at room temperature. ATO methanol solution were added into TC-PEG-PLGA solution (ATO: TC-PEG-PLGA = 10%, w/w). The mixed solution was dialyzed (MWCO 7.0kDaa) to against pure water for 24 h with frequent exchanges of pure water to remove methanol. After dialysis, the mixed solution was centrifuged at 4000 rpm for 10 minutes to remove unencapsulated ATO, and the TC-PEG-PLGA/ATO micelles were obtained.

### Characterization of TC-PEG-PLGA/ATO micelles

The morphological examinations were performed using a Hitachi-7700 transmission electron microscopy (TEM, Hitachi, Tokyo, Japan). The samples were dropped on copper grids and stained with 2% (w/v) phosphotungstic acid for viewing. Samples were stored at 4 or 37 °C for different periods of time, and the size and polydispersity index (PDI) were measured using dynamic light scattering (DLS, Brookhaven Instruments, Holtsville, NY).

The content of ATO was assayed by HPLC method with C18 column (250 mm × 4.6 mm, 5 μm). Acetonitrile (0.02 mol l^−1^) and acetic acid solution (65:35, V/V) were used as the mobile phase. The column temperature and the detection wavelength were set as 40 °C and 249 nm with flow rate at 1.0 mL min^−1^ (Chunjian, 2012). The drug loading (DL%) and encapsulation efficiency (EE%) were measured using an ultrafiltration tube with a molecular weight cutoff of 30 kDa. Unencapsulated ATO in the substrate was assayed by HPLC method. DL% and EE% were calculated using the following equations:

EE(%)=weight of ATO in micelles/weight of feeding ATO×100%

DL(%)=weight of encapsulated ATO in micelles/total weight of micelles × 100%

### *In vitro* drug release

*In vitro* release profiles of TC-PEG-PLGA/ATO micelles were investigated using the dialysis method. Briefly free ATO, PEG-PLGA/ATO micelles and TC-PEG-PLGA/ATO micelles were loaded into the dialysis bag (MWCO 7.0 kDa) and immersed into 50 mL PBS (pH 7.4). The experiments were performed under horizontal shaking (60 rpm) at 37 °C. At pre-determined time intervals, samples were withdrawn and replaced with fresh medium. The content of ATO was measured using HPLC method. All drug-release tests were repeated thrice.

### Cytotoxicity study

The cytotoxicity of TC-PEG-PLGA was evaluated using the MTT assay. The MC3T3-E1 cells were seeded in a 96-well plate at 5 × 10^3^ cells/well and incubated for 24 h, respectively. After that, cells were exposed to PEG-PLGA micelles and TC-PEG-PLGA micelles with serial concentrations for 48 h. Afterwards, 15 μL of 3-(4,5-dimethylthiazol-2-yl)-2,5-diphenyltetrazolium bromide (MTT) was added to each well and incubated further for another 4 h at 37 °C. The media were removed and 200 μl of DMSO was added into each well to dissolve MTT formazan. After shaken for 20 min, the absorbance of formazan at 570 nm was measured with a microplate reader. The tests were repeated thrice.

### Cellular uptake of TC-PEG-PLGA micelles

DiD fluorescent probe (excitation wavelength/emission wavelength 644/663 nm) was used to label TC-PEG-PLGA micelles, and both TC-PEG-PLGA/DiD and PEG-PLGA/DiD micelles were prepared by the solvent diffusion method. MC3T3-E1 cells were seeded in 12-well plates at 1 × 10^5^ ml^−1^ cells/well, and incubated for 24 h. Then, the cells were treated with a fresh medium containing TC-PEG-PLGA/DiD micelles or PEG-PLGA/DID micelles (equal DiD) for further incubation. At predetermined intervals (2 and 4 h), the cells were washed with PBS and then observed using a confocal microscopy (Olympus, Japan). In addition, cellular uptake of TC-PEG-PLGA/DiD and PEG-PLGA/DiD micelles was assessed using flow cytometry, and the tests were performed in triplicates.

### Bone mineral binding ability *in vitro*

To investigate the bone mineral binding ability between bone-like substrates and bone-targeted delivery system *in vitro*, hydroxyapatite (HAp) assay was performed according to the previous report (Ryu et al., 2016). The initial fluorescence emission intensities of TC-PEG-PLGA and PEG-PLGA micelles (5 mL, 0.1 wt.%) were detected via fluorospectrophotometry and then separately added into PBS containing HAp (10 mg ml^−1^) and shaken for 3 h in the dark. After filtrated with 0.22 μm microporous membrane, the supernatant intensity after HAp adsorption was detected. HAp adsorption affinity was calculated using the following equations:

$$\text{HAp adsorption affinity}=\;\left({\text{I}}_{\text{initial}}\text{intensity}\;-\;{\text{I}}_{\text{supernatant}}\text{intensity after HAp adsorption}\right)/{\text{I}}_{\text{initial}}\text{intensity}$$

### Alizarin red S assay

MC3T3-E1 cells were seeded in 24-well plates at 1 × 10^4^  ml^−1^ cells/well and were cultured in osteogenic differentiation medium containing 50 μg ml^−1  ^L-ascorbic acid and 10 mmol l^−1^ β-glycerophosphate. MC3T3-E1 cells were treated with free ATO, TC-PEG-PLGA/ATO or PEG-PLGA/ATO micelles (equal ATO) for 14 days, respectively and the medium was refreshed every other day. After 14 days, the cells were fixed with 10% formalin and then stained with Alizarin Red S. Matrix mineralization was quantified by incubating the samples in 100 mM cetylpyridinium chloride for 2 h. The alizarin red S concentration was measured at 570 nm (Wang et al., 2014). The tests were repeated thrice.

### Biodistribution of TC-PEG-PLGA micelles

To investigate bone-targeted ability of TC-PEG-PLGA micelles, ICR mice were injected intravenously with Dir-loaded TC-PEG-PLGA or PEG-PLGA micelles (5 μg Dir per rat). 24 h after administration the mice were sacrificed, and their organs (heart, liver, spleen, lung and kidney) and femur were collected. Imaging was performed using the *In vivo* Imaging System (DXS4000PRO, Kodak, Rochester, NY), respectively.

### Pharmacokinetic study

Female Sprague Dawley rats with a mean body weight of 180–200 g were fasted for 12 h before the experiment, with access to water. The experimental protocols and animal care were approved by the Committee for Animal Experiments of Traditional Chinese Medicine University of Guangzhou. In this study, the rats were randomly divided into three groups (*n* = 5). Free ATO solution (0.5% tween), PEG-PLGA/ATO micelle and TC-PEG-PLGA/ATO micelle solution with an equivalent dose of ATO were intravenously injected at a dose of 2.5 mg kg^−1^. Blood samples (500 μL) were withdrawn from orbit at predetermined times (0.5, 1, 2, 4, 6, 8, 12, 24, 36 and 48 h), and immediately put into heparinized micro tubes. The obtained blood samples were centrifugation at 4000 rpm for 10 min, and then stored at −20 °C until analysis by HPLC. To determine ATO concentration, 150 μl plasma was mixed with 850 μl methanol solution and vortexed for 10 min, and then centrifuged for 15 min at 10,000 rpm. The supernatant was transferred and evaporated under the nitrogen flow. The extraction residual was re-dissolved in the mobile phase solution and injected for analysis. The analysis was performed on Agilent-C18 column (250 mm × 4.6 mm, 5 μm) with a security guard column (C18, 10 × 4 mm, 5 mm); mobile phase: Acetonitrile/0.02 mol l^−1^ and acetic acid solution (65:35, V/V); detection wavelength: 249 nm; flow rate: 1.0 mL min^−1^; column temperature: 40 °C; injection volume: 20 μL (Chunjian, 2012). The linear standard curve presented good linearity over the concentration range of 5–1000 ng ml^−1^. The standard curve in plasma: *A* = 45.8 C + 8.243 (R^2^ = 0.9992). The method specificity, precision and recovery rate met the requirements of methodology.

### Pharmacodynamic study

Female Sprague Dawley rats with a mean body weight of 180–200  g were fasted for 12 h before the experiment, with access to water. The pharmacodynamics of TC-PEG-PLGA/ATO were assessed using an ovariectomy (OVX)-induced osteoporosis model. The experiment groups included five groups: (1) Sham group; (2) OVX group; (3) OVX group treated with ATO; (4) OVX group treated with PEG-PLGA/ATO micelles; (5) OVX group treated with TC-PEG-PLGA/ATO micelles. Injectable solutions were prepared before administration as a solution of each treatment dose in physiological saline. The experimental groups were subjected to intravenous administration of each solution (0.2 mL every two days). After 12-week treatment, rats were sacrificed for pharmacodynamic evaluation. The left femurs were isolated and determined for assessing bone mineral density (BMD) using dual-energy X-ray absorptiometry. The right femora were used to determine bone mechanical strength (BMS) using mechanical testing machine.

### Statistical analysis

The results were expressed as mean ± standard deviations. Pharmacokinetic data were analyzed using the Drug and Statistics Software (DAS 2.0). The statistical analysis was carried out by using Student’s *t*-test and the statistical significance was designated as *p* < 0.05.

## Results and discussion

### Synthesis and characterization of TC-PEG-PLGA conjugates

**Figure 1.**
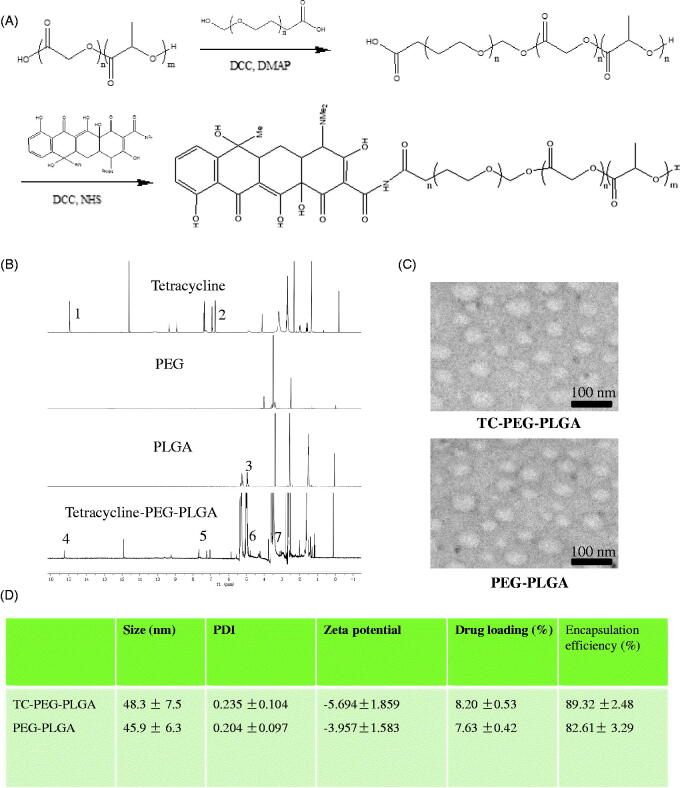
Preparation and characterization of TC-PEG-PLGA. (A) Synthesis route of TC-PEG-PLGA. (B) ^1^H NMR spectra. (C) Negative-stain transmission electron microscopy of TC-PEG-PLGA/ATO and PEG-PLGA/ATO micelles. (D) Encapsulation efficiency and drug loading of ATO-loaded micelles.

TC-PEG-PLGA was successfully synthesized via two steps of chemical reaction, including esterification reaction between hydroxy group of PEG and carboxyl group of PLGA and then amide reaction between carboxyl group of PEG and amino group of TC (Figure 1). The structure of TC-PEG-PLGA was confirmed using ^1^H NMR spectrum. The ^1^H NMR spectra of TC, PEG, PLGA and TC-PEG-PLGA are showed in Figure 1(B). The characteristic peaks of TC (1 and 2) are observed in H- NMR spectrum of TC-PEG-PLGA (4 and 5). In addition, the characteristic peak of PLGA (3) is also observed in the spectrum of TC-PEG-PLGA (6). Based on this, it is evidence that the TC-PEG-PLGA has been successfully synthesized.

The synthesized TC-PEG-PLGA in aqueous medium could self-assemble to form micelles. The CMC value is an important characteristic for amphiphilic materials and represents the self-assembly ability to form micelles. The CMC values of TC-PEG-PLGA and PEG-PLGA used as the control were determined via fluorescence method using pyrene as a probe. The CMC of TC-PEG-PLGA and PEG-PLGA conjugates at room temperature were 19.4 and 17.5 μg ml^−1^ respectively, which indicated that two conjugates had good capacity to form micelles with a core-shell structure in aqueous medium. PLGA as hydrophobic core could be used to store hydrophobic drugs and increase the drug solubility. PEG as a hydrophilic shell could enhance micellar stability in aqueous medium. Benefiting from low toxicity and no immunogenicity, both PLGA and PEG have large development potential as drug carrier.

### Preparation and characterization of ATO-loaded TC-PEG-PLGA micelles

The TC-PEG-PLGA/ATO micelles were prepared through solvent diffusion method. Drug encapsulating efficiency of TC-PEG-PLGA/ATO and PEG-PLGA/ATO micelles were 89.32 ± 2.48 and 82.61 ± 3.29%, and drug loading were 8.20 ± 0.53 and 7.63 ± 0.42% during 10% drug feeding amount (Figure 1(D)). The obtained TC-PEG-PLGA/ATO micelles were evaluated by TEM and DLS. Figure 1(C) shows that TC-PEG-PLGA/ATO and PEG-PLGA/ATO micelles had a uniform spherical shape and their sizes were 48.3 ± 7.5 and 45.9 ± 6.3 nm, respectively. The particle size of ATO-loaded micelles was smaller than blank micelles (56.3 ± 6.9 and 54.5 ± 8.3 nm) which was associated with the hydrophobic interaction between the hydrophobic chains (PLGA) and free ATO becoming stronger after ATO loading. In addition, the size difference may be due to the different conditions at which the DLS detect the hydrodynamic micelle size while TEM observes the dried state.

**Figure 2.**
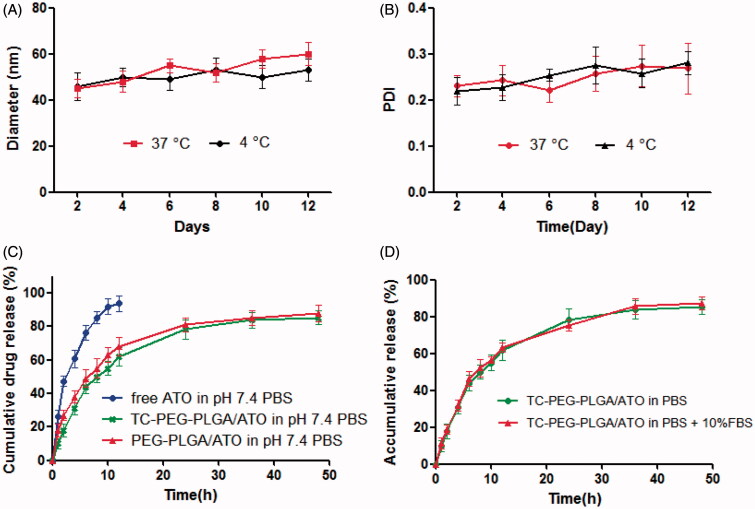
*In vitro* stability of TC-PEG-PLGA/ATO micelles at 4 °C or 37 °C. (A) Size and (B) PDI (mean ± SD, *n* = 3). (C) *In vitro* release profiles of free ATO, TC-PEG-PLGA/ATO and PEG-PLGA/ATO micelles at 37 °C in pH 7.4 PBS (mean ± SD, *n* = 3). (D) *In vitro* release profiles of TC-PEG-PLGA/ATO micelles at 37 °C in pH 7.4 PBS and pH 7.4 PBS containing 10% fetal bovine serum (mean ± SD, *n* = 3).

To investigate *in vitro* stability of TC-PEG-PLGA/ATO micelles at 37 and 4 °C, micellar size and PDI were detected at different periods of time (2, 4, 6, 8, 10 and 12 d). Figure 2(A,B) shows that micellar size remains nearly unchanged within 12 d at 4 °C in comparison to 37 °C and PDI increases slightly over the same period, which provides the strong evidence that TC-PEG-PLGA/ATO micelles can keep good colloidal stability at 4 °C.

### *In vitro* ATO release from TC-PEG-PLGA/ATO micelles

*In vitro* ATO release of TC-PEG-PLGA/ATO micelles in pH 7.4 PBS were investigated by dialysis method. As shown in Figure 2(C), free ATO is released rapidly, more than 90% of it within 12 h. In contrast, sustained release of ATO from micelles can be maintained for more than 48 h. TC-PEG-PLGA/ATO micelles show a biphasic release pattern, including fast release during the initial time (0–12 h) and slow release later on (12–48 h). Approximately 40% ATO is released from TC-PEG-PLGA/ATO micelles during the first 12 h and the rest of ATO was released during the late time intervals (12-48 h). Moreover, PEG-PLGA/ATO micelles show the similar release behavior, which indicates that the modified TC has no effects on drug-release behaviors. *In vitro* drug release behavior of TC-PEG-PLGA/ATO micelles was also evaluated in pH 7.4 PBS containing 10% fetal bovine serum (10% FBS, Figure 2(D)). No significant difference in ATO release was observed between thetwo dissolution media (*p* > 0.05).

### Cytotoxicity

**Figure 3.**
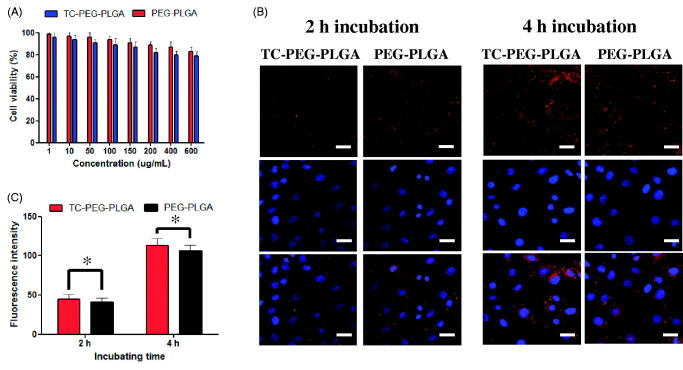
*In vitro* study of TC-PEG-PLGA micelles. (A) Viability of MC3T3-E1 cells after exposure to PEG-PLGA micelles or TC-PEG-PLGA (mean ± SD, *n* = 3). (B) Fluorescence images of MC3T3-E1 cells were incubated with DiD-loaded PEG-PLGA and TC-PEG-PLGA micelles for 2 and 4 h, respectively (scale bar =25 mm). (C) Quantitative results of cellular uptake measured by flow cytometry. *p > 0.05.

Cytotoxicity of MC3T3-E1 cells (osteoblastic cells) was minimally affected by incubation with PEG-PLGA micelles and TC-PEG-PLGA micelles even at the highest concentration tested. As presented in Figure 3(A), TC-PEG-PLGA displays minimum cytotoxicity with over 80% MC3T3-E1 cells remaining viable at the highest concentration of 600 μg ml^−1^. The result indicates that TC-PEG-PLGA developed in this work is minimally cytotoxic which is essential for drug delivery carrier.

### Cellular uptake study

The cellular uptake test of TC-PEG-PLGA micelles was investigated in MC3T3-E1 cells. In this study, fluorescent probe DiD was used to label TC-PEG-PLGA micelles. Figure 3(B) presents the cellular images of MC3T3-E1 cells after incubation with TC-PEG-PLGA/DiD or PEG-PLGA/DiD for 2 and 4 h, respectively. The results show that both TC-PEG-PLGA/DiD and PEG-PLGA/DiD can be internalized into MC3T3-E1 cells in a time-dependent manner. Moreover, the cellular uptake of TC-PEG-PLGA micelles was quantitatively assessed using flow cytometry. As presented in Figure 3(C), fluorescence intensity inside MC3T3-E1 cells incubated with TC-PEG-PLGA or PEG-PLGA micelles show no significant difference at 2 and 4 h (*p* > 0.05), which demonstrates that the conjugated TC has no effects on micellar internalization.

### HAp affinity

**Figure 4.**
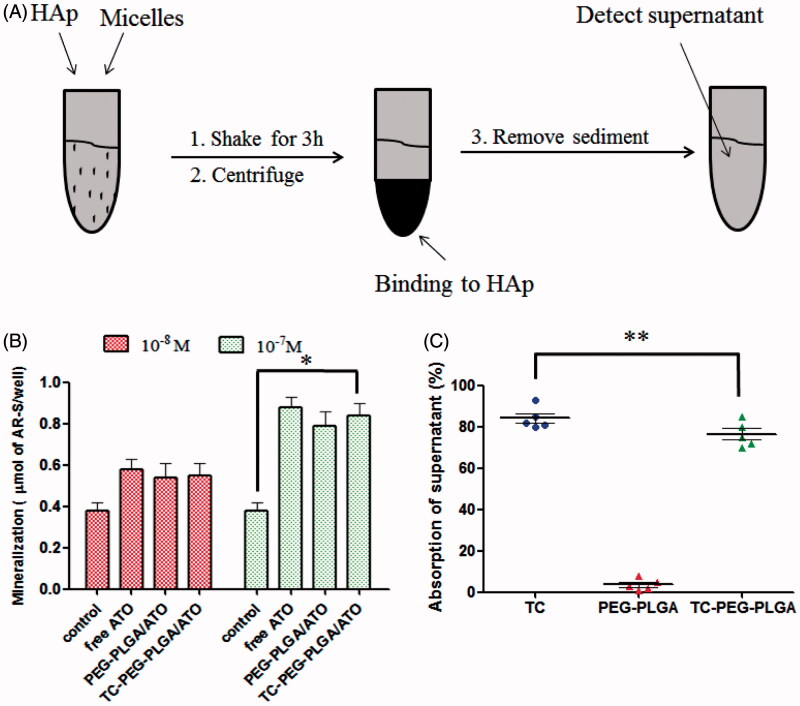
*In vitro* targeted efficacy and pharmacodynamic results. (A) and (B) Schematic diagram of HAp adsorption affinity experiment using FITC as a drug model and their results (mean ± SD, *n* = 5). (C) Effects of TC-PEG-PLGA/ATO micelles on mineralization of extracellular matrix by MC3T3-E1 cell culture (mean ± SD, *n* = 5). *p < 0.05, **p > 0.05.

The circulation times of ATO encapsulated in micelles can be prolonged but is not sufficient enough to delivery to the target sites. High binding activity to bone tissue is a prerequisite for bone-targeted delivery. Therefore, the affinity of TC-PEG-PLGA micelles to HAp was quantitatively analyzed using HAp binding assay. Figure 4(A) shows the binding ratio of TC, PEG-PLGA and TC-PEG-PLGA to HAp. Most TCbound to the HAp powder (84.8%) whereas only a small amount of PEG-PLGA (4.8%) are non-specifically adsorbed. TC-PEG-PLGA shows a lower binding ratio (77.3%) than that of TC, which is associated with the larger size of TC-PEG-PLGA micelles than that of TC molecules. The results demonstrates that TC-PEG-PLGA micelles had good binding affinity to bone tissue, despite lower binding ratio than free TC.

### Effects on mineralized nodule formation

The effects of TC-PEG-PLGA/ATO micelles on osteoblast differentiation were assessed by quantitative mineralization. MC3T3-E1 cells were cultured with osteogenic differentiation medium containing TC-PEG-PLGA/ATO or PEG-PLGA/ATO micelles (equal ATO) for 14 days. Given the osteogenic effects of osteogenic differentiation medium containing 50 μg ml^−1^ ascorbic acid and 10 mM β-glycerophosphate,differentiation medium without micelles was used as background treatment. As shown in Figure 4(C), the increased cell mineralization can be observed in group cultured with free ATO, PEG-PLGA/ATO and TC-PEG-PLGA/ATO micelles in comparison to control. Moreover, the mineralization effect of ATO shows dose-dependent manner.

### Biodistribution of TC-PEG-PLGA micelles

**Figure 5.**
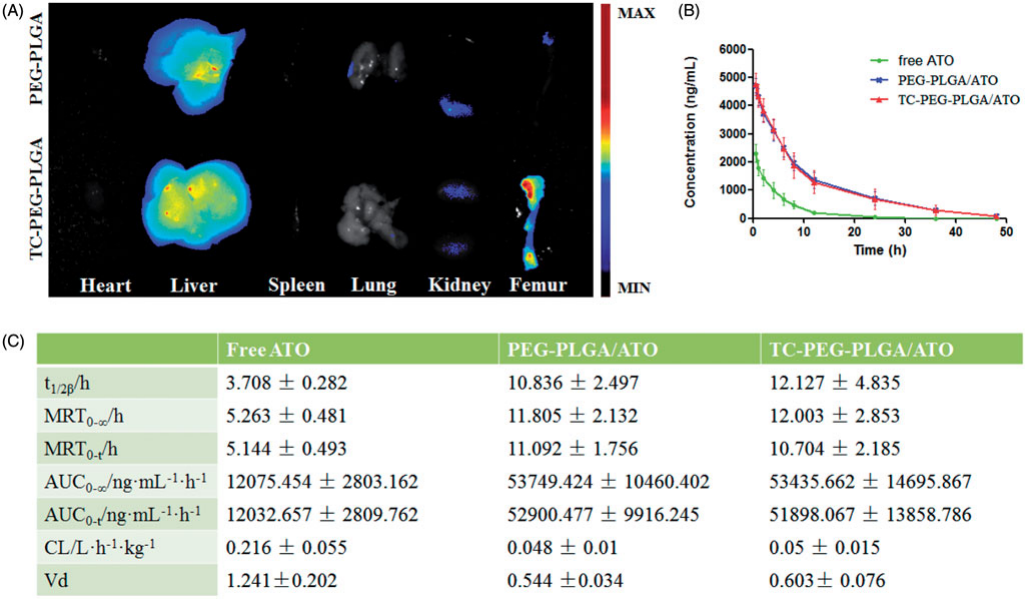
Biodistribution and pharmacokinetic study. (A) Images of dissected tissues at 24 h, including heart, liver, spleen, lung, kidney and bone. (B) and (C) Pharmacokinetics of free ATO, PEG-PLGA/ATO or TC-PEG-PLGA/ATO micelles in rats (mean ± SD, *n* = 5). T_1/2_: half-life; AUC: area under the concentration-time curve; MRT: mean residence time; CL: clearance rate; V_d_: apparent volume of distribution.

To further verify the bone-targeting efficacy of TC-PEG-PLGA micelles, near-infrared fluorescent probe Dir-loaded micelles were intravenously injected in ICR mice. At 24 h after administration, mice were sacrificed and their organs (heart, liver, spleen, lung and kidney) and femur were collected. As shown in Figure 5(A), femur in the mouse receiving TC-PEG-PLGA/Dir micelles exhibits strong fluorescence in comparison to that receiving PEG-PLGA/Dir micelles, although much micelles are inevitably accumulated in the liver. The result of biodistribution is also consistent with HAp binding assay that TC-mediated micelles can bond to the HAp powder at higher density than PEG-PLGA micelles. Therefore, biodistribution result demonstrates the promising potential of TC-PEG-PLGA micelles for bone-targeted delivery.

### Pharmacokinetics of TC-PEG-PLGA/ATO micelles in healthy rats

To investigate whether TC-PEG-PLGA micelles could effectively shield ATO leakage from micelles and prolong the circulation time, pharmacokinetic profiles of TC-PEG-PLGA/ATO micelles were determined in healthy rats, free ATO and PEG-PLGA/ATO micelles were used as control. The mean ATO plasma concentration time profiles after intravenous administration in different formulation, free ATO solution (0.5% tween), PEG-PLGA/ATO micelle and TC-PEG-PLGA/ATO micelle solution are presented in Figure 5(B). Figure 5(C) summarizes the relevant pharmacokinetic parameters. As shown in Figure 5(B), free ATO plasma concentration reduces quickly due to rapid distribution and metabolism, leading to short t_1/2_, approximately 3.708 h. Little ATO is detected in the plasma at 12 h after administration. In contrast, appreciable ATO can still be detected in rats treated with TC-PEG-PLGA/ATO micelles at 24 h after administration, leading to the prolonged t_1/2_, 12.127 ± 4.835 h. Compared with free ATO, the area under concentration curve (AUC_0-∞_) in TC-PEG-PLGA/ATO micelle group is significantly enhanced from 12075.454  ± 2803.162 ng ml^−1^ h^−1^ to 53435.662 ± 14695.867 ng ml^−1^ h^−1^. The mean residence time (MRT) enhances from 5.263 ± 0.481 h to 12.003 ± 2.853 h after ATO encapsulated in TC-PEG-PLGA micelles, suggesting that micelles can prolong circulation time of ATO through reducing its rapid elimination and contributing to more ATO distributed into bone tissue. All these findings suggest that TC-PEG-PLGA micelles can remain having a good integrity and stability during systemic circulation and prevent ATO being metabolized and eliminated as far as possible. In addition, no significant difference in pharmacokinetic profiles, including t_1/2β_, MRT, AUC, CL and V_d_, were observed between TC-PEG-PLGA/ATO micelles and PEG-PLGA/ATO micelles (*p* > 0.05) which indicates that TC has no effects on the pharmacokinetic behaviors of micelles.

### Anti-osteoporosis of TC-PEG-PLGA/ATO micelles

**Figure 6.**
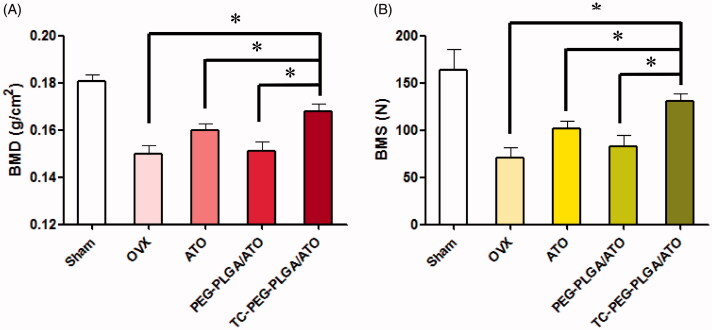
Anti-osteoporosis of TC-PEG-PLGA/ATO micelles. (A) Bone mineral density (BMD) of the femur in osteoporotic rats after 12 weeks treatment (mean ± SD, *n* = 5). (B) Bone mechanical strength (BMS) of femur in osteoporotic rats after 12 weeks treatment (mean ± SD, *n* = 5). *p < 0.05.

Osteoporosis therapy (OST) aims to decreasing the fracture risk by enhancing bone strength, a critical parameter driven by BMD. BMD is extensively used as a predictor for evaluating fracture risk and measured using dual energy X-ray absorptiometry in the clinical practice (Ammann & Rizzdi, 2003). In this study, osteoporotic rats were induced by ovariectomy operation and fed for one month. The experiment groups included five groups: (1) Sham group; (2) OVX group; (3) OVX group treated with ATO; (4) OVX group treated with PEG-PLGA/ATO micelles; (5) OVX group treated with TC-PEG-PLGA/ATO micelles. The BMD of femur in OVX groups was significantly different in comparison to those in sham group. The BMD of femur in sham group were 0.179 ± 0.008 g cm^2^, whereas BMD in OVX group were 0.151 ± 0.008 g cm^2^, which showed the successful induction of osteoporosis model in OVX rats. After 12 weeks treatment, the rats were sacrificed and BMD was determined via dual energy X-ray absorptiometry. As shown in Figure 6(A), the femur BMD in OVX group receiving pharmacological intervention and TC-PEG-PLGA/ATO micelles show the best therapeutic efficacy in comparison to free ATO and PEG-PLGA/ATO micelles (0.167 ± 0.008 g cm^2^ vs 0.159 ± 0.007 g cm^2^ and 0.151 ± 0.009 g cm^2^, *p < 0.05).

Figure 6(B) presents the effect of TC-PEG-PLGA/ATO micelles on BMS of femurs. BMS in the normal rats is 189 ± 18.8 N and decreases to 72.6 ± 20 N in OVX rats. After pharmacological therapy the BMS is significantly increased in TC-PEG-PLGA/ATO micelle group in comparison to free ATO group and PEG-PLGA/ATO micelle group (129.8 ± 23 vs 100.4 ± 22 and 80.4 ± 29, *p* < 0.05).

Overall, pharmacodynamic results demonstrate the good therapeutic effects of TC-PEG-PLGA/ATO micelles in osteoporotic rats due to increased ATO in bone tissue as showed in BMD and BMS. In addition, targeted delivery system can moderately reduce the required ATO and then decrease ATO-induced side effects in other organs. In this pharmacodynamic evaluation BMD and BMS are determined to assess therapeutic outcomes in osteoporosis because they are badly needed. Hip fracture is closely related with quality of life and mortality rate, especially in elderly population (Johnell et al., 2004).

## Conclusion

In this study, we successfully developed a novel amphiphilic TC-PEG-PLGA, which spontaneously self-assembles into stable micelles in aqueous medium. Upon formulation with hydrophobic ATO, the TC-PEG-PLGA/ATO micelles demonstrate active targeting to the bone tissue in osteoporotic rats, as evidenced by the alteration of ATO pharmacokinetics and biodistribution results. Upon further optimization, this ATO-loaded delivery system may have the potential to be translated into clinical applications in managing osteoporosis.

## References

[CIT0001] Ammann P, Rizzoli R. (2003). Bone strength and its determinants. Osteoporos Int 14:S13–S18. 12730800 10.1007/s00198-002-1345-4

[CIT0002] Aukrust P, Yndestad A, Smith C, et al. (2005). Potential role for immunomodulatory therapy in atherosclerotic plaque stabilisation. Expert Opin Pharmacother 6:2169–80. 16218879 10.1517/14656566.6.13.2169

[CIT0003] Chan WW, Wong GT, Irwin MG. (2013). Perioperative statin therapy. Expert Opin Pharmacother 14:831–42. 23521372 10.1517/14656566.2013.782003

[CIT0004] Chuengsamarn S, Rattanamongkoulgul S, Suwanwalaikorn S, et al. (2010). Effects of statins vs. non-statin lipidlowering therapy on bone formation and bone mineral density biomarkers in patients with hyperlipidemia. Bone 46:1011–15. 20045497 10.1016/j.bone.2009.12.023

[CIT0005] Chunjian Z. (2012). Determination of Atorvastatin in Human Plasma by HPLC and Pharmacokinetics Study. China Pharmacy 23:2833–5.

[CIT0006] Davignon J, Mabile L. (2001). Mechanisms of action of statins and their pleiotropic effects. Ann Endocrinol (Paris) 62:101–12. 11240412

[CIT0007] Du YZ, Weng Q, Yuan H, et al. (2010). Synthesis and antitumor activity of stearate-g-dextran micelles for intracellular doxorubicin delivery. ACS Nano 4:6894–902. 20939508 10.1021/nn100927t

[CIT0008] Esposito K, Capuano A, Sportiello L, et al. (2013). Should we abandon statins in the prevention of bone fractures? Endocrine 44:326–33. 23526261 10.1007/s12020-013-9924-z

[CIT0009] Hamrick I, Schrager S, Nye AM. (2015). Treatment of osteoporosis: current state of the art. Wien Med Wochenschr 165:54–64. 25502850 10.1007/s10354-014-0335-4

[CIT0010] Johnell O, Kanis JA. (2004). An estimate of the worldwide prevalence, mortality and disability associated with hip fracture. Osteoporos Int 15:897–902. 15490120 10.1007/s00198-004-1627-0

[CIT0011] Lips P, Jameson K, Bianchi ML, et al. (2009). Working group for quality of life of the international osteoporosis foundation. Osteoporos Int 21:61–70.19504036

[CIT0012] Luhmann T, Germershaus O, Groll J, et al. (2012). Bone targeting for the treatment of osteoporosis. J Control Release 161:198–213. 22016072 10.1016/j.jconrel.2011.10.001

[CIT0013] Moshiri A, Oryan A, Meimandi-Parizi A,et al. (2014). Effectiveness of xenogenous-based bovine-derived platelet gel embedded within a threedimensional collagen implant on the healing and regeneration of the Achilles tendon defect in rabbits. Expert Opin Biol Ther 14:1065–89. 24840092 10.1517/14712598.2014.915305 PMC4743604

[CIT0014] Nicolas J, Mura S, Brambilla D, et al. (2013). Design, functionalization strategies and biomedical applications of targeted biodegradable/biocompatible polymer-based nanocarriers for drug delivery. Chem Soc Rev 42:1147–235. 23238558 10.1039/c2cs35265f

[CIT0015] Oryan A, Alidadi S, Moshiri A, et al. (2014). Bone regenerative medicine: classic options, novel strategies, and future directions. J Orthop Surg Res 9:18–25. 24628910 10.1186/1749-799X-9-18 PMC3995444

[CIT0016] Oryan A, Moshiri A, Meimandi Parizi A. (2012). Alcoholic extract of Tarantula cubensis improves sharp ruptured tendon healing after primary repair in rabbits. Am J Orthop 41:554–60. 23431525

[CIT0017] Patel J, Martin SS, Banach M. (2016). Expert opinion: the therapeutic challenges faced by statin intolerance. Expert Opin Pharmacother 17:1497–507. 27254275 10.1080/14656566.2016.1197202

[CIT0018] Perrin DD. (1965). Binding of tetracyclines to bone. Nature 208:787–8. 5868891 10.1038/208787a0

[CIT0019] Ryu TK, Kang RH, Jeong KY, et al. (2016). Bone-targeted delivery of nanodiamond-based drug carriers conjugated with alendronate for potential osteoporosis treatment. J Control Release 232:152–60. 27094604 10.1016/j.jconrel.2016.04.025

[CIT0020] Shirke SS, Jadhav SR, Jagtap AG. (2008). Methanolic extract of Cuminum cyminum inhibits ovariectomy-induced bone loss in rats. Exp Biol Med (Maywood) 233:1403–10. 18824723 10.3181/0803-RM-93

[CIT0021] Shuid AN, Ibrahim N, Mohd Amin MC, et al. (2013). Drug delivery systems for prevention and treatment of osteoporotic fracture. Curr Drug Targets 14:1558–64. 24200294 10.2174/1389450114666131108153905

[CIT0022] Wang CZ, Fu YC, Jian SC, et al. (2014). Synthesis and characterization of cationic polymeric nanoparticles as simvastatin carriers for enhancing the osteogenesis of bone marrow mesenchymal stem cells. J Colloid Interface Sci 432:190–9. 25086394 10.1016/j.jcis.2014.06.037

